# Exploring atomic defects in molybdenum disulphide monolayers

**DOI:** 10.1038/ncomms7293

**Published:** 2015-02-19

**Authors:** Jinhua Hong, Zhixin Hu, Matt Probert, Kun Li, Danhui Lv, Xinan Yang, Lin Gu, Nannan Mao, Qingliang Feng, Liming Xie, Jin Zhang, Dianzhong Wu, Zhiyong Zhang, Chuanhong Jin, Wei Ji, Xixiang Zhang, Jun Yuan, Ze Zhang

**Affiliations:** 1 State Key Laboratory of Silicon Materials, Key Laboratory of Advanced Materials and Applications for Batteries of Zhejiang Province, School of Materials Science and Engineering, Zhejiang University, Hangzhou, Zhejiang 310027, China; 2 Beijing Key Laboratory of Optoelectronic Functional Materials and Micro-Nano Devices, Department of Physics, Renmin University of China, Beijing 100872, China; 3 Department of Physics, University of York, Heslington, York YO10 5DD, UK; 4 Advanced Nanofabrication, Imaging and Characterization Core Lab, King Abdullah University of Science and Technology (KAUST), Thuwal 239955, Kingdom of Saudi Arabia; 5 Instituteof Physics, Chinese Academy of Sciences, c/o Collaborative Innovation Center of Quantum Matter, Beijing 100190, China; 6 CAS Key Laboratory of Standardization and Measurement for Nanotechnology, National Center for Nanoscience and Technology, Beijing 100190, China; 7 Center for Nanochemistry, Beijing National Laboratory for Molecular Sciences, Key Laboratory for the Physics and Chemistry of Nanodevices, State Key Laboratory for Structural Chemistry of Unstable and Stable Species, College of Chemistry and Molecular Engineering, Peking University, Beijing 100871, China; 8 Key Laboratory for the Physics and Chemistry of Nanodevices and Department of Electronics, Peking University, Beijing 100871, China; 9 Department of Physics and Astronomy, Collaborative Innovation Center of Advanced Microstructures, Shanghai Jiao Tong University, Shanghai 200240, China

## Abstract

Defects usually play an important role in tailoring various properties of two-dimensional materials. Defects in two-dimensional monolayer molybdenum disulphide may be responsible for large variation of electric and optical properties. Here we present a comprehensive joint experiment–theory investigation of point defects in monolayer molybdenum disulphide prepared by mechanical exfoliation, physical and chemical vapour deposition. Defect species are systematically identified and their concentrations determined by aberration-corrected scanning transmission electron microscopy, and also studied by *ab-initio* calculation. Defect density up to 3.5 × 10^13^ cm^−2^ is found and the dominant category of defects changes from sulphur vacancy in mechanical exfoliation and chemical vapour deposition samples to molybdenum antisite in physical vapour deposition samples. Influence of defects on electronic structure and charge-carrier mobility are predicted by calculation and observed by electric transport measurement. In light of these results, the growth of ultra-high-quality monolayer molybdenum disulphide appears a primary task for the community pursuing high-performance electronic devices.

## Abstract (web summary)

Imperfections can greatly alter a material’s properties. Here, the authors investigate the influence of point defects on the electronic structure, charge-carrier mobility and optical absorption of molybdenum disulphide prepared by mechanical exfoliation, physical and chemical vapour deposition.

## Graphical abstract

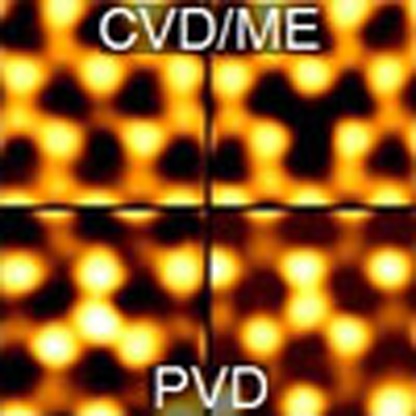

The success of graphene^1^,^2^ offers a paradigm for the exploration of novel low-dimensional physical phenomena^3^ and physical properties in two-dimensional (2D) crystal systems^4^,^5^. However, its intrinsic shortcoming lies in its zero bandgap, which strongly hinders its application in logical electronic devices. Among the post-graphene development, 2D semiconducting molybdenum disulphide and other transition metal dichalcogenides^6^,^7^ have recently appeared on the horizon of materials science and condensed matter physics. Monolayer MoS_2_ is a direct-gap semiconductor that exhibits a substantially improved efficiency in its photoluminescence^8^,^9^. Valley polarization occurs due to significant spin–orbit coupling and leads to optical circular dichroism^10^,^11^,^12^ in the monolayer system. The electronic transport of MoS_2_-based field effect transistors (FETs) shows steep sub-threshold swing of 70 mV dec^−1^ (refs 13, 14, 15) and a high on/off ratio up to 10^8^ (ref. 16). A metal-insulator transition happens when carrier densities reaches 10^13^ cm^−2^, which also increases the effective mobility^17^,^18^,^19^. Owing to its unique optical and electric properties, MoS_2_ is believed to be a promising candidate as a building block for future applications in nanoelectronics and optoelectronics^6^.

Wafer-scale production of atomically thin layers is paramount for MoS_2_ to be used as a candidate channel material for electronic and optoelectronic devices^13^,^14^,^15^,^16^,^17^,^18^,^19^. Among the currently available preparation methods, mechanical exfoliation (ME) is deemed less efficient for these large-scale applications, even though it produces the highest-quality samples exhibiting the best electric performance. Physical and chemical vapour deposition^17^,^20^,^21^,^22^,^23^,^24^,^25^ methods are more compatible for the scalable growth of high-quality samples. However, the experimentally attainable mobility is still one or two order-of-magnitude lower than the theoretical value of 410 cm^2^ V^−1^ s^−1^ (refs 13, 18, 26, 27, 28, 29). For back-gated m-MoS_2_ FET devices, the highest mobility reported so far reaches 81 cm^2^ V^−1^ s^−1^ for ME sample^28^, 45 cm^2^ V^−1^ s^−1^ for chemical vapour deposition (CVD)^17^ and<1 cm^2^ V^−1^ s^−1^ for physical vapour deposition (PVD)^25^. The major scattering mechanism for the mobility deterioration has been recently suggested as due to the presence of plentiful localized band tail states^30^ caused by short-range disordered structural defects (such as vacancies^28^,^31^ and grain boundaries^32^), and Coulomb traps^33^,^34^. However, the roles played by various defects in electric and optoelectronic properties are yet to be explicitly understood. There have been few investigations reported on point defects, mostly vacancies, and grain boundaries in m-MoS_2_ (refs 28, 30, 31, 32, 35, 36). These studies are, however, often performed with samples made by preparatory methods, for example, ME or CVD, which essentially limits the scope of those studies.

Here we present a systematic investigation of the point defects in distinctly prepared m-MoS_2_ by combining atomically resolved annular dark-field scanning transmission electron microscopy (ADF-STEM) imaging, density functional theory (DFT) calculation and electric transport measurements. We observe, for the first time, that antisite defects with molybdenum replacing sulphur are dominant point defects in PVD-grown MoS_2_, while the sulphur vacancies are predominant in ME and CVD specimens. These experimental observations are further supported qualitatively by the growth mechanism and quantitatively by the defects’ formation energies calculations. The DFT calculations, in addition, predict the electronic structures and magnetic properties of m-MoS_2_ with antisite defects. We also discuss the influence of defects on the phonon-limited carrier mobility theoretically, and further examine them by electric transport in defective m-MoS_2_-based FETs. Our systematic investigation of point defects, especially antisites, will further deepen our understanding of this novel 2D atomically thin semiconductor and pave the way for the scalable electronic application of the family of atomically thin transition metal chalcogenides.

## Results

### Statistics of point defects

**Figure 1:**
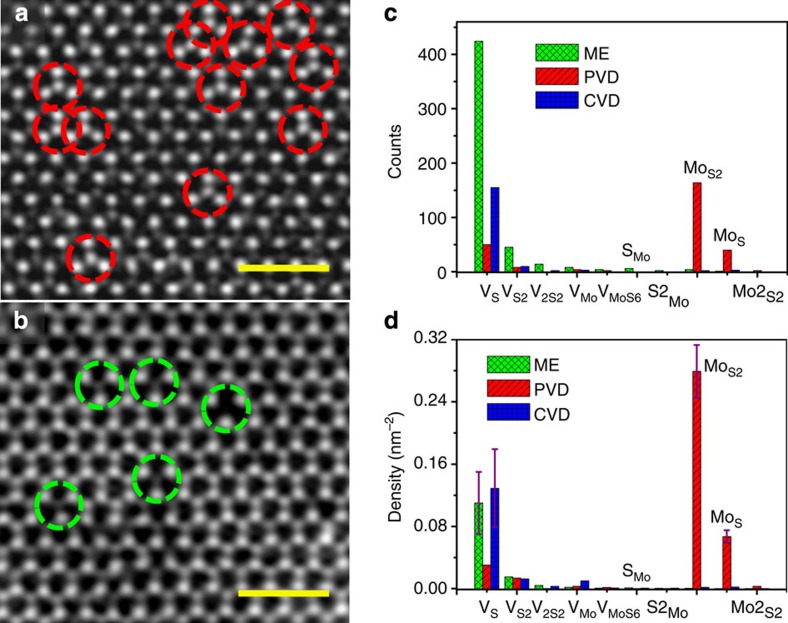
Atomic resolved STEM–ADF images to reveal the distribution of different point defects. (**a**) Antisite defects in PVD MoS_2_ monolayers. Scale bar, 1 nm. (**b**) Vacancies including V_S_ and V_S2_ observed in ME monolayers, similar to that observed for CVD sample. Scale bar, 1 nm. (**c**,**d**) Histograms of various point defects in PVD, CVD and ME monolayers. Error estimates are given for the dominant defects (more details on the statistics can be found in Supplementary Fig. 6). ME data are in green, PVD data in red and CVD in blue.

For the purpose of the analysis of defects and their concentration, we have chosen about ten samples prepared under the optimized fabrication condition (see the Methods section and Supplementary Note 1 for the details of sample synthesis) from each method (ME, PVD and CVD) and then transferred each sample onto at least two TEM grids independently for ADF-STEM characterizations. The crystalline quality and the choice of samples for statistical analysis are presented in Supplementary Figs 1–9. Figure 1 summarized the most significant results obtained from our analysis on these MoS_2_ samples. For the PVD specimen, antisite defects with one Mo atom replacing one or two S atoms (Mo_S_ or Mo_S2_) are frequently observed, marked with red dashed circles shown in Fig. 1a, while the dominant defects for the ME and CVD samples are S vacancies with one (V_S_) or two (V_S2_) S atoms absent, as marked by green dashed circles in Fig. 1b. As the STEM’s *Z*-contrast mechanism^37^, that is, *I*~*Z*^1.6–2.0^ (*I* and *Z* are the image contrast and atomic number, respectively), predicts, Mo and S atoms can be unambiguously discriminated, with Mo (*Z*=44) showing bright contrast and two superposed S atoms showing dim contrast in the lattice of m-MoS_2_. Following a similar argument and quantitative image analysis, various defects, for example, Mo_S_ or V_S_ where the lattice image presents abnormal intensity variation, can be clearly identified individually through direct imaging and their atomic structures further verified by *ab-initio* calculations (please refer to Supplementary Table 1 for details of each atomic defect).

We show the relative importance of each type of point defects in Fig. 1c,d. Figure 1c presents the total counts of different point defects based on over 70 atomically resolved ADF-STEM images for each type, that is, ME, PVD or CVD MoS_2_ samples. It is found that the dominant type of point defects in each sample highly depends on the specific sample preparation method. The V_S_ vacancy is the predominant point defects in ME and CVD samples, with its concentration of about (1.2±0.4) × 10^13^ cm^−2^ (Supplementary Fig. 6), close to the results reported previously^30^. Atomic defects V_Mo_ (one Mo atom missing) and S_Mo_ (one S atom replacing Mo site) were also found, but with much lower concentrations as shown in Fig. 1c. In contrast, the histogram also shows that antisite defects Mo_S2_ and Mo_S_ are dominant in PVD samples, with their concentrations higher than that of V_S_. The density of Mo_S2_ and Mo_S_ reaches (2.8±0.3) × 10^13^ and 7.0 × 10^12^ cm^−2^, corresponding to an atomic percent of 0.8% and 0.21%, respectively (counted on the total number of all Mo and S atoms). Such a defect concentration is surprisingly high if the defects were regarded as impurity doping, which is usually only achieved in degenerate semiconductor^38^ (for instance 10^−2^~10^−4^). It is, therefore, of vital importance to understand how they modify the electronic properties of m-MoS_2_ as elucidated below.

### Structural characterization of point defects

**Figure 2:**
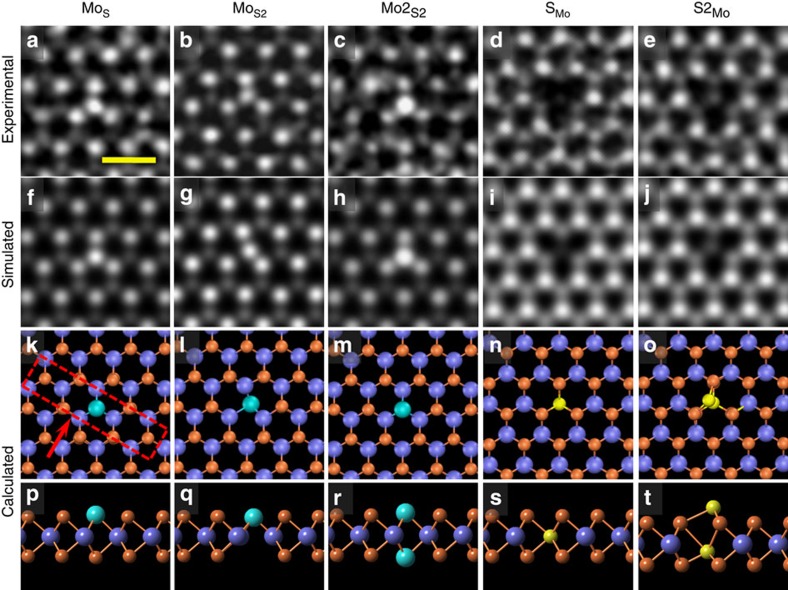
Atomic structures of antisite defects. (**a**–**c**) High-resolution STEM–ADF images of antisite Mo_S_, Mo_S2_ and 
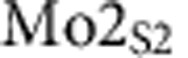
, respectively. The former two antisites (highlighted by the red dashed rectangle in **k**) are dominant in PVD-synthesized MoS_2_ single layers. Scale bar, 0.5 nm (**d**,**e**) Atomic structures of antisite defects S_Mo_ and 
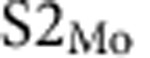
, respectively. (**f**–**j**) Simulated STEM images based on the theoretically relaxed structures of the corresponding point defects in (**a**–**e**), using simulation software QSTEM^49^. (**k**–**t**) Relaxed atomic model of all antisite defects in **a**–**e** through DFT calculation, with top and side views, respectively. Light blue, Mo atoms; gold, S atoms. For ease of comparison, we have presented the simulated ADF images before the atomistic schematics of the DFT calculated structures.

So far, there have been few reports concerning the structures of sulphur vacancies^31^,^39^ and their impacts on the electronic transport properties^30^,^40^ of m-MoS_2_; by contrast, there is still a lack of detailed knowledge on the antisite defects, which is at such an unexpected high doping level in PVD samples. Hence, we focus more on antisite defects. In Fig. 2a–e, we highlight all the images of the experimentally observed antisite defects in m-MoS_2_ (see also Supplementary Fig. 10), which can be grouped into two categories. One is the antisite defects with Mo atom(s) substituting S atom(s), including Mo_S_, Mo_S2_ and 
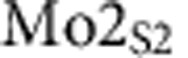
 (Fig. 2a–c). The other category is the antisite defects with S atom occupying the site of Mo, namely S_Mo_ and 
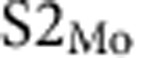
 (Fig. 2d,e). The experimental identification of these antisite defects can be further unambiguously supported by the quantitative image simulation based on DFT-predicted atomic structures of all antisite defects. The fully relaxed DFT-predicted atomic structures of antisite defects were shown in Fig. 2k–t, where the atomic displacement and structure deformation are explicitly observable, especially for antisites Mo_S2_ and 
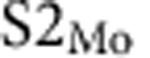
. Associated side views of these relaxed structures are available in Fig. 2p–t. The ADF image simulations (Fig. 2f–j) based on the calculated structures fit quite well with the experimental images shown in Fig. 2a–e, respectively, especially for the off-centre feature observable in antisites Mo_S2_ and 
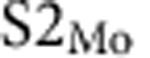
, which can be tentatively attributed to Jahn–Teller distortions.

### Energetics of predominant point defects in different m-MoS_2_

The distribution of different atomic defects in m-MoS_2_ certainly depends on their preparation process. A full exploration of the growth dynamics requires a comprehensive experiment–theory joint investigation, which is beyond the scope of the present work, although it is of fundamental interest. Here we provide a qualitative explanation, based on our DFT calculations, to reveal the microscopic physical mechanism of the preparation-process-dependent defect formation. The formation energy (Δ*E*_Form_) for all the point defects are calculated and summarized in Table 1, as consistent with a previous report^35^. Here, chemical potentials of elements Mo and S were employed to calculate the formation enthalpy (Δ*H*_Form_) of defects. To account for a range of different possible reservoirs (for example, bulk element or bulk MoS_2_), each enthalpy is given with a range, as listed in Table 1. Two widely used DFT codes (CASTEP^41^ and VASP (Vienna *Ab-initio* Simulation Package)^42^) are adopted to exploit the full range of functionality available and demonstrate the consistency of the calculated results in Table 1.

An ME sample is exfoliated from MoS_2_ natural mineral. After the MoS_2_ mineral was formed and/or extracted, either element S or Mo of MoS_2_ is prone to reach a solid–gas phase equilibrium. Owing to a higher saturated vapour pressure of S, the mineral-form MoS_2_ has to release more S than Mo atoms into the gas phase and thus S is prone to be deficient in MoS_2_. Reflecting this fact, vacancies V_S_ and V_S2_ have the lowest Δ*E*_Form_ of 2.12 eV and 4.14 eV, respectively, among all the defects. The formation energies of all the antisite defects are higher than 5 eV, indicating that the S-deficient mineral-form MoS_2_ favours the formation of S vacancies, leading to the observation of the most common defect of V_S_, followed by V_S2_, and almost no antisite defect in ME samples.

In a typical PVD process, MoS_2_ precursor is sublimated into the gas phase with clusters and atoms, carried by Ar gas (mixed with H_2_), and then condensed into a solid-phase MoS_2_. Sulphur has a larger saturated vapour pressure so that more S atoms in the gas phase will leave the preparation chamber, thus establishing a S-deficient and Mo-rich condition. These clusters and atoms are highly mobile and are thus prone to form an ordered structure of MoS_2_ in the lowest total energy. Considering *n*+1 Mo and 2*n*-1S atoms for example, they have two options, namely, forming (i) *n* MoS_2_ units with one Mo_S_ antisite or (ii) *n*+1 MoS_2_ units with three V_S_ vacancies. The exact value of *n* does not affect the energetic difference between these two types of defects. We thus arbitrarily instantiate *n* as 107, namely 108 Mo and 213 S atoms in total. The total energy of the antisite option is −1,774.83 eV, while that for the vacancy case is −1,774.26 eV which is 0.57 eV less stable than the former. A similar relation also applies to antisite defect Mo_S2_ with an energy gain of 0.92 eV. A sample with one antisite Mo_S_ or Mo_S2_ shares the same number of Mo and S atoms with another sample that has three or four S vacancies, respectively. The formation energies of antisites Mo_S_ and Mo_S2_ were, therefore, divided by three and four, respectively, to make these energies quantitatively comparable with a single S vacancy, as required by the comparison with Boltzmann distribution. We have renormalized Δ*E*′_Form_(Mo_S_)=1.93 eV, Δ*E*′_Form_(Mo_S2_)=1.89 eV, which give rise to a ratio of *p*(Mo_S2_):*p*(Mo_S_):*p*(V_S_)=10.5:6.7:1 at the growth temperature of 1,100 K. This ratio is comparable with the experimental probability density ratio of Mo_S2_:Mo_S_:V_S_=9:2.3:1 in PVD samples.

The CVD process is distinctly different from ME or PVD. Extra S vapour is supplied to replace O in the MoO_3_ precursor under an S-rich condition. We suspect that there are small amount of residual O atoms taking the position of S atoms in the resulting MoS_2_ sheets, due to the competition between Mo–O and Mo–S bonding in the reaction chamber. Our *ab-initio* calculation, not shown here, suggests that these O atoms are 1.99 eV less stable than corresponding 2S and usually tend to desorb into the gas phase leaving vacancies at the S sites, that is, S vacancies. It is argued that Mo atoms may jump into the S vacancies and form Mo antisites. Despite of the S-rich condition, even if Mo is rich in a certain local environment, Mo atoms may be firmly bonded with oxygen in the precursor, which strongly limits the diffusion of Mo, making the formation of Mo antisite from mobile Mo atom and S vacancy much less likely.

### Electronic structures

**Figure 3:**
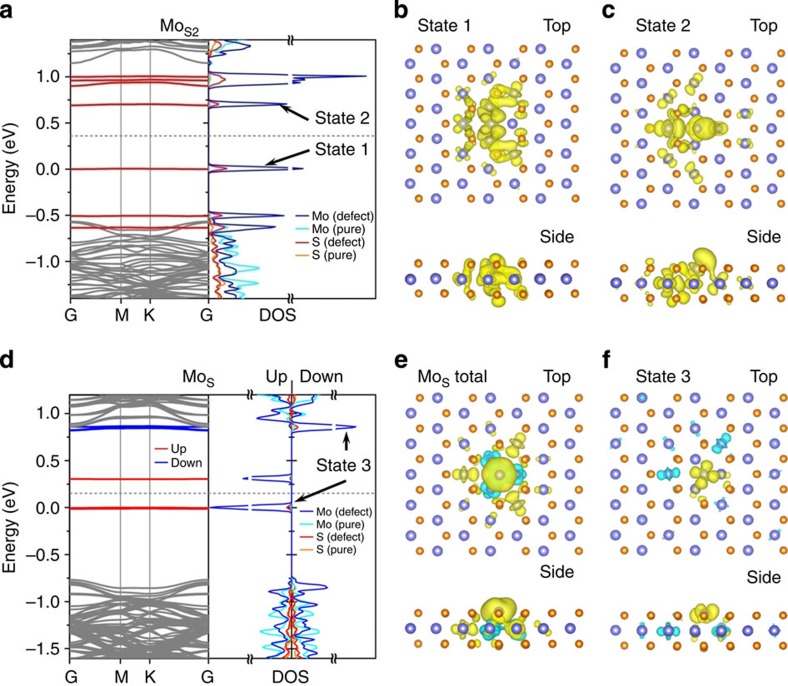
Electronic properties of predominant antisite defects in MoS_2_ monolayer. (**a**) Band structure and corresponding density of states (DOS) of antisite defect Mo_S2_.The grey bands are from normal lattice sites, similar to conduction band and valence band of perfect monolayer, while the discrete red bands show the localized defects states. The DOS is projected onto the atoms around the defect (defect) and those in the middle plane of two adjacent defects (pure), respectively. The grey dash line indicates the position of the Fermi Level. (**b**,**c**) Real-space distribution of the wave functions of the two defect states below and above the Fermi energy. (**d**) The band structure and DOS of antisite Mo_S_, with a similar colour scheme of **a**, but the two spin components are coloured in red (spin-up) and blue (spin-down), respectively. (**e**) Spin density of antisite Mo_S_, defined as *ρ*_up_−*ρ*_down_, charge densities *ρ*_up_ and *ρ*_down_ are spin-resolved for spin-up and -down components, which are represented by yellow and blue isosurfaces, respectively. (**f**) Spin-resolved real-space distribution of the wave function of the two marked defect states (State 3) in **d**. The isosurface value in **b**,**c**,**e**,**f** is 0.001*e* Bhor^−3^.

The electronic structure of point defects plays a crucial role in determining the electric properties of these defective m-MoS_2_. Vacancy V_S_ and its effect on electronic structures have been recently reported^30^,^35^; we thus focus on the less-studied antisite defects (please refer to Supplementary Fig. 11 for our ADF imaging and DFT calculation of V_S_). Figure 3 shows the theoretically predicted band structures and projected density-of-states of two primary antisites Mo_S_ and Mo_S2_. Our results give a bandgap of 1.73 eV for a defect-free m-MoS_2_ (Supplementary Fig. 12), close to the experimentally observed optical bandgap of 1.8 eV (ref. 8). Defect states with nearly flat band dispersion for Mo_S2_ and Mo_S_ reside inside the band gap of a perfect m-MoS_2_ (Fig. 3a,d). These states mostly comprises the *d* orbitals of four Mo atoms around the defect. In addition, the orbital hybridization of Mo and S atoms results in extended wavefunctions involving the surrounding atoms, forming a ‘superatom’ with a radius of roughly 6 Å, as shown in Fig. 3b,c,f.

Magnetic properties of m-MoS_2_ have not been reported yet, as it is believed to be a non-magnetic material. Nevertheless, we did find a local magnetic moment of 2 *μ*_B_ in antisite Mo_S_, while the values for other defects, for example, V_S_, V_S2_ and Mo_S2_, are smaller than 0.1 *μ*_B_, and hence are negligible. The magnetic moment of 2 *μ*_B_ for a Mo_S_ antisite is not localized only on the central Mo atom, with the surrounding atoms contributing roughly 20% of the total moment probably due to the strong hybridization among these atoms in a ‘superatom’, as shown in the visualized total spin density (Fig. 3e). Detailed distribution of magnetic moment is available in Supplementary Fig. 13. The spin-resolved real-space distribution of a defect-induced state (state 3) marked in Fig. 3d was plotted in Fig. 3f to illustrate the origin of the magnetism. The occupied spin-up component (yellow isosurface) is mainly composed of the *d*_xy_ and *d*_x2–y2_ orbitals of the antisite Mo atom, while the unoccupied spin-down component (cyan isosurface) is projected onto the *d*_xy_ and *d*_z2_ orbitals of surrounding Mo atoms, consistent with the total spin charge density shown in Fig. 3e. More detailed discussion on the magnetic property of antisites are presented in Supplementary Fig. 14 and Supplementary Note 2.

### Carrier mobility in defective samples

**Figure 4:**
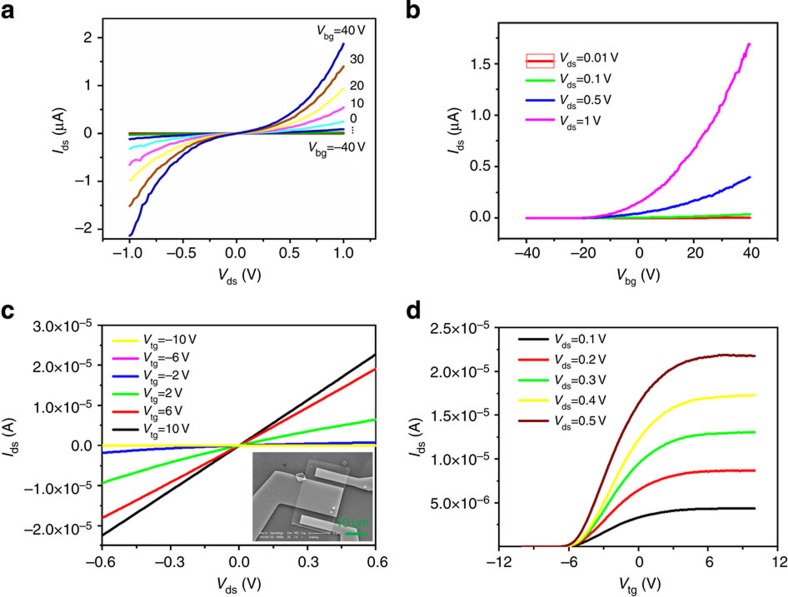
Electric transport of defective MoS_2_. (**a**,**b**) Output and transfer characteristics of PVD MoS_2_-based FET. (**c**,**d**) Output and transfer characteristics of CVD MoS_2_-based FET.

As there have been plentiful reports on the transport of ME MoS_2_-based FETs with electron mobility 1~81 cm^2^ V^−1^ s^−1^ (refs 15, 16, 18, 26, 28, 43, 44), we focus on the transport properties of CVD and PVD monolayers. Figure 4a–d presents the output and transfer characteristics of fabricated FETs based on PVD and CVD MoS_2_, respectively. Our transport measurements (in Fig. 4a–d) of defective MoS_2_-based FETs reveal that the PVD and CVD MoS_2_ has electron mobility 0.5 and 11 cm^2^ V^−1^ s^−1^, respectively. All these results are well comparable with the reported mobilities of m-MoS_2_ of 1~81 cm^2^ V^−1^ s^−1^ for ME^15^,^16^,^18^,^26^,^28^,^43^,^44^, 5~45 cm^2^ V^−1^ s^−1^ for CVD^17^,^29^ and the reported values of <1 cm^2^ V^−1^ s^−1^ for PVD m-MoS_2_ (ref. 25) respectively.

Theoretically, we focus on the effect of the defects on the phonon-limited carrier mobilities^45^,^46^,^47^. Table 2 lists the calculated effective masses, deformation potentials and estimated mobilities derived based on the predicted electron mobility of 410 cm^2^ V^−1^ s^−1^ in a perfect m-MoS_2_ (ref. 48). MoS_2_ samples are usually n-type, we thus primarily focus on the electron mobility. It is found that the phonon-limited mobility of electrons flowing in the intrinsic conduction band is, exceptionally, nearly unaffected by the presence of vacancies (V_S_ or V_S2_), but reduced by three times in the samples with antisite defects, whereas the phonon-limited mobility of holes carried by the intrinsic valence band is more sensitive to these defects and reduces roughly three times for vacancy and more than four times for antisite. Both vacancy and antisite are strong electron-scattering centres that the mobility derived from the defect states (d–e and d–h) for either electron or hole is fairly small, mostly smaller than 1 and 10 cm^2^ V^−1^ s^−1^, respectively. The defect states strongly affect, but not overwhelmingly dominate, the overall carrier mobility of the samples, owing to the relative low density of defects and the strongly localized defect states. On the other hand, in a real FET device the measured mobility can be affected by the contact resistance^45^ or the carrier density^19^. Furthermore, the trapped charges would act as a scattering centre^15^,^40^. A hopping transport caused by localized disorder is also observed^30^,^33^. Both can effectively reduce the mobility of the device. Nevertheless, our theory shows that the measured mobility is, most likely, correlated with the primary type of defects in a sample.

## Discussion

We have to address the possible influence of electron beam irradiation on the formation of atomic defects and to distinguish the native from irradiation-induced defects. The observed antisite defects are believed to be native, not caused by electron beam irradiation. We envisage that two steps are involved in the formation of a Mo_S_ defect from a well-prepared sample, namely the formation of a V_S_ vacancy and Mo adatom, followed by the capture of the Mo adatom by the S vacancy. Although sulphur vacancy could be created by electron beam sputtering^31^,^39^, the formation of a Mo adatom and adjacent Mo vacancy need substantially high energy transfer from electron irradiation, which is less likely. The *in-situ* experiments show that the Mo adatoms are very mobile, but rarely jump into S vacancies to form antisite defects (Supplementary Note 3 and Supplementary Figs 15 and 16). On considering these experimental and simulation results, the observed Mo_S_ and Mo_S2_ antisites should be confidently regarded as intrinsic defects. In terms of S vacancies, as suggested by early studies^30^,^31^, the concentration of sulphur vacancies may be slightly overestimated due to beam damage even if the microscope works at low accelerating voltage (Supplementary Fig. 17).

MoS_2_ sheets are extensively adopted in electronic devices. These point defects, as localized disorders, are significant scattering centres of carriers, which may reduce the mobility of charge carriers through the intrinsic conduction or valence band, especially for samples with antisite defects. Therefore, growth of ultra-high-quality m-MoS_2_ is of crucial importance to fabricate high-performance electronic devices. On the other side, the presence of defects may provide us novel routes to tailor the properties of m-MoS_2_. We predicted theoretically for the first time that antisite Mo_S_ shows a magnetic moment of 2 *μ*_B_. Our prediction of local magnetic moments may promote further investigations on the magnetic properties of defective MoS_2_ monolayers. Given the strong optoelectronic response of MoS_2_ layers with Mo_S_ defects, it is a probable material that is capable for optical manipulation of local magnetic moment. If the defect density goes sufficiently high, it may expect an appreciably large magnetic exchange interaction between defects, and thus become a promising model system for the studies of dilute-magnetic semiconductors and 2D magnetism.

Based on these findings, we propose an application-oriented strategy for fabricating atomically thin MoS_2_. In terms of electric applications, extra S should be introduced into the growth process or post-growth treatment, to restrain the formation of antisite defects for PVD specimen or to heal the abundant S vacancies for CVD and ME specimens, whereas in respect to magnetism, varied pressure of S in the PVD growth could produce different densities of Mo_Sx_ antisites that remain to be explored for magnetic applications.

In summary, our systematic investigation of geometric and electronic structures of antisites and vacancies of m-MoS_2_ by ADF-STEM imaging and DFT calculation has led to a considerable progress in our understanding of the variation of the electric and magnetic properties induced by these point defects. We have demonstrated that minimizing point defects, especially antisites, is paramount for electric transport applications, while controllably introduced antisites may produce atomic size local magnetic moments. All these results considerably improve the understanding of point defect in atomically thin transition metal dichalcogenides and should benefit their potential applications in optoelectronic and nanoelectronic devices.

## Methods

### Sample preparations and transfer

ME m-MoS_2_ was prepared by micro-cleavage^8^ of natural bulk crystal (SPI Supplies) using scotch tapes. The monolayer was identified from the optical contrast of thin flakes under an optical microscope (Zeiss A2m) and then transferred onto copper TEM grids covered with holey carbon films.

CVD monolayers were synthesized through the reduction of precursor MoO_3_ by sulphur vapour flow at ambient pressures following the previously reported method^21^,^32^. PVD MoS_2_ monolayers used in this study were synthesized by thermal evaporation of MoS_2_ powders (Sigma-Aldrich, 99%) at a temperature of 950 °C. Ar (2 s.c.c.m.) and H_2_ (0.5 s.c.c.m.) were used as the carriers gases, following the reported method in ref. 25. The pressure of the growth chamber was about 8 Pa and the growth time was usually 10 min. In terms of sample synthesis, the advantages and disadvantages of these methods and liquid-phase exfoliation are compared in Supplementary Table 2.

PVD and CVD MoS_2_ monolayers were transferred onto the TEM grid as follows: first, the SiO_2_ substrates with monolayer samples were covered with polymethyl methacrylate (PMMA) film after spin coating and then dried in air at 120 °C for 5 min. The substrates were immersed into the boiling sodium hydroxide solution (1 mol l^−1^), which was heated up to 200 °C to etch away the underneath SiO_2_ layers. The floating PMMA film was picked up with a clean glass slide and then transferred into the distilled water for several cycles to wash away surface residues. In the next step, the PMMA film was lifted out by a TEM grid covered with lacey carbon film and then dried naturally in ambient. This TEM grid was heated at 120 °C for 5 min in air before immersion into hot acetone for about 24 h, to remove the PMMA. Before the ADF-STEM characterization, all the monolayer specimens on TEM grids were annealed at 200 °C in air for 10 min to reduce surface residues and/or contaminations.

### STEM characterization and image simulation

Most of the structural characterizations of m-MoS_2_ were carried out with a probe-corrected Titan ChemiSTEM (FEI, USA). We operated this microscope at an acceleration voltage of 80 kV to alleviate specimen damage induced by beam radiation. A low probe current was selected (<70 pA) and the convergence angle was set to be 22 mrad. Under such a condition, the probe size was estimated to be close to 1.5 Å. To enhance the contrast of the sulphur sublattices, the so-called medium-range ADF mode rather than the high-angle ADF mode was used by adjusting the camera length properly. Some experiments (such as Figs 1a and 2a–c) were done with an ARM 200CF (JEOL, Japan), equipped with a cold field-emission gun. The advantage of higher energy resolution (0.3 eV) and smaller probe size (<1.2 Å) provides higher resolution, thus giving rise to sharper contrast of the atomic images. All the experimental images shown in the main text and Supplementary Information were filtered through the standard Wiener deconvolution to partially remove the background noise for a better display (Supplementary Fig. 10).

It should be noted that intrinsic adatom defects were seldom observed experimentally and, therefore, they are not considered here. All the image statistics were done on the clean regions of the examined samples by ADF–STEM. On considering the good homogeneity of these samples prepared under the optimized conditions (see Supplementary Information), it shall not lead any large variations in the analysed defect population.

STEM–ADF image simulations of relaxed antisite defects were done by software QSTEM^49^. The input parameters were set according to the experimental conditions. Probe size, convergence angle and acceptance angle of the ADF detector are critical and accounted for in the image simulation.

### DFT calculations

The defect formation enthalpy for the first column of Table 1 was calculated using the total energy method with the plane-wave pseudopotential DFT code CASTEP^41^. The basic methodology is well known and has been widely used before for defect calculations. In this study, the Perdew-Burke-Ernzerhof-generalized gradient approximation^50^ with ultrasoft potentials is used, as supplied in the CASTEP library. In addition, the dispersion interactions were added using the semi-empirical scheme of Grimme^51^. Structural optimizations of both ionic positions and cell vectors are performed using a modified Broyden-Fletcher-Goldfarb-Shanno-like scheme. The calculations were performed in a slab geometry of a 6 × 6 supercell of m-MoS_2_ with a 15 Å vacuum space in the *c* axis direction perpendicular to the monolayer. A comparative study of the defect formation enthalpy and energy, together with the electronic and magnetic properties were also done by VASP simulation code^42^ using the same slab model. The projector augmented-wave method^52^ combined with a plane wave basis is adopted in the calculations. The energy cutoff for plane wave is 400 eV in structural relaxation and increases to 500 eV while calculating the energy and electronic properties. The optB86b exchange functional^53^ together with the vdW correlation^54^,^55^ was adopted for exchange-correlation functional. The Brillouin zone of the supercell is sampled by a 3 × 3 × 1 *k*-mesh. All these structures are fully relaxed until the residual force for each atom is less than 0.02 eV Å^−1^.

### Estimation of formation energy and enthalpy

The formation energy was defined as, Δ*E*_Form_=*E*_System_–*N*_S_ × *E*_S_ML_–*N*_Mo_ × *E*_Mo_ML_, where *E*_S_ML_=*E*_S(single)_+*E*_Bond_, *E*_Mo_ML_=*E*_Mo(single)_+2*E*_Bond_ and *E*_Bond_=(*E*_ML_–*E*_Mo(single)_−2*E*_S(single)_)/3. Formation enthalpy of defects is defined as 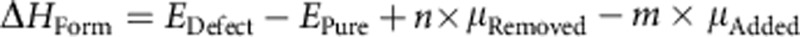, where *μ*_Removed_ and *μ*_Added_ are the chemical potentials of the removed and added atoms to form a defect, respectively. Chemical potentials of Mo and S in MoS_2_ fulfill the equation 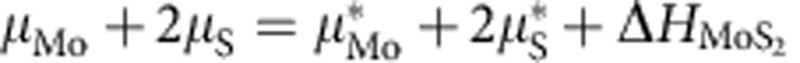, where μ*_Mo_ is the chemical potential of Mo in the bulk form, μ*_S_ is the chemical potential of S in the α-phase crystal form and 
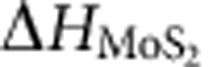
is the formation enthalpy of MoS_2_. Although it is difficult to obtain the exact values of *μ*_Mo_ and *μ*_S_, the range of them can be deduced as 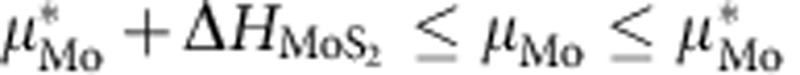, 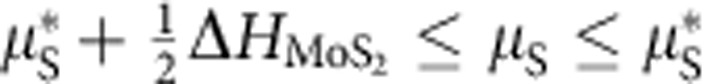.

There are two formation enthalpy values in Table 1, the former one was computed by choosing *μ*_Mo_ and *μ*_S_ equal to μ*_Mo_ and μ*_S_, respectively, indicating that the removed (added) atoms come from (go to) the pure bulk form of Mo and S. For the latter value, we set 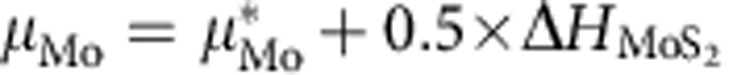 and 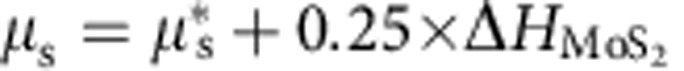. In this case, the source and drain of defect atoms are pure MoS_2_ ML. For antisite defects Mo_S2_ and 
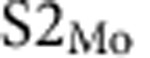
, both schemes give the same result.

### Estimation of ‘phonon-limited’ carrier mobility

In 2D, the carrier mobility is given by the expression^45^,^46^,^47^


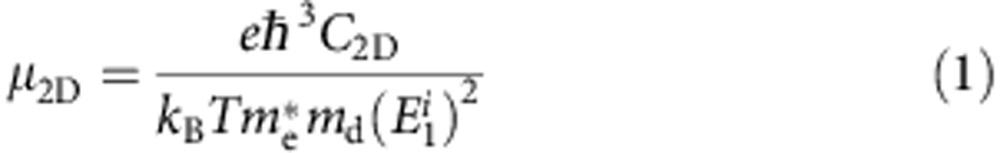


where 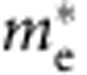 is the effective mass in the transport direction and *m*_*d*_ is the average effective mass determined by 
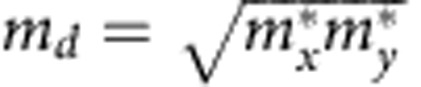
. The term *E*_1_ represents the deformation potential constant of the valence-band maximum for holes or conduction-band minimum for electrons along the transport direction, defined by 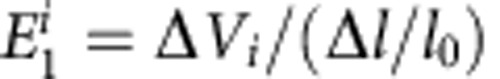. Here Δ*V*_i_ is the energy change of the *i*^th^ band under proper cell compression and dilatation, *l*_0_ is the lattice constant in the transport direction and Δ*l* is the deformation of *l*_0_.

### FET fabrication and transport

The monolayer MoS_2_-based FET devices are fabricated through the following process. First, source (S) and drain (D) electrodes of the devices were defined via e-beam lithography and a 5/45 nm Ti/Au film was then evaporated followed by a standard lift-off process. In addition, the back-gated MoS_2_ FETs were then finished. Second, the top-gated devices were begun with forming gate insulator. Gate oxide layer (30 nm HfO_2_ film) was grown under 90 °C through Atomic Layer Deposition (Cambridge NanoTech Inc.). Lastly, the gate electrode window was also defined by e-beam lithography, followed by evaporation of 5 nm Ti and 45 nm Au thin film, and the top-gated MoS_2_ FETs are finished after a lift-off process. The as-fabricated devices were measured through Keithley 4200 semiconductor analyser on a probe station at room temperature and in air. An example of the device architecture is shown in Supplementary Fig. 18.

## Author contributions

J.H. and Z.H. contributed equally to this work. C.J., J.Y. and W.J. conceived the research. J.H., D.L. and N.M. contributed to the sample preparations. J.H. and C.J did most of the STEM characterizations, with the assistance from K.L., X.Y., L.G. and X.Z., J.H., J.Y. and C.J. were responsible for the STEM data analysis and image simulations. Z.H., W.J. and M.P. did the DFT calculations. N.M., L.X. and J.H. contributed to the synthesis, measurement and analysis of PL spectra and the PVD devices of PVD samples. D.W. and Z.Z. contributed to the FET device fabrications and electric transport measurements on CVD samples. All authors discussed the results and contributed to the preparation of the manuscript.

## Additional information

**How to cite this article:** Hong, J. *et al*. Exploring atomic defects in molybdenum disulphide monolayers. *Nat. Commun.* 6:6293 doi: 10.1038/ncomms7293 (2015).

## Supplementary Material

Supplementary Information Supplementary Figures 1-18, Supplementary Tables 1-2, Supplementary Notes 1-3 and Supplementary References

## Figures and Tables

**Table 1:** Formation energy (Δ*E*_Form_) and enthalpy (Δ*H*_Form_) of considered point defects.

	**CASTEP**	**VASP**	
	***ΔH***_**Form**_**(eV)**	**Δ*****H***_**Form**_**(eV)**	***ΔE***_**Form**_**(eV)**
Mo_S_	6.22~7.29	5.45~6.09	5.79
Mo2_S2_	11.15	7.95	7.54
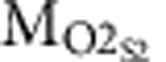	—	9.81~11.09	10.49
S_Mo_	6.65~5.58	6.11~5.47	5.77
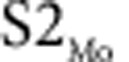	8.00	7.09	7.49
V_S_	2.74~1.67	2.86~2.22	2.12
V_S2_	—	5.63~4.34	4.14
V_Mo_	6.98~4.84	7.28~5.99	6.20

CASTEP, Cambridge Sequential Total Energy Package; VASP, Vienna *Ab-initio* Simulation Package.

The formation enthalpy is defined as *ΔH*_Form_=*E*_Defect_–*E*_Pure_+*n* × *μ*_Removed_–*m* × *kμ*_Added_*. μ* is the chemical potential of the removed and/or added atom to form a defect, while the formation energy is defined as*ΔE*_Form_=*E*_System_–*N*_S_ × *E*_S_ML_–*N*_Mo_ × *E*_Mo_ML_, where *E*_S_ML_ and *E*_Mo_ML_ are the single atom energy of Mo and S in a perfect monolayer. (Please refer to the Methods section for more details).

Different exchange-correlation functionals are used in the VASP and CASTEP codes as discussed in the text.

**Table 2:** Phonon-limited carrier mobility estimation of perfect and defective MoS_2_ monolayers.

	m*/***m***_**0**_				***E*****(eV)**				***μ*** **(cm**^**2**^**V**^**−1**^**s**^**−1**^)			
	**e**	**h**	**d-e**	**d-h**	**e**	**h**	**d-e**	**d-h**	**e**	**h**	**d-e**	**d-h**
Perfect	0.40	−0.57	—	—	−14.9	−3.4	—	—	410	3850	—	—
V_S_	0.43	−0.96	35.2	105.6	−13.9	−3.4	−4.5	5.4	410	1390	<1	<1
V_S2_	0.42	−1.2	31.1	9.2	−13.9	−3.1	−4.9	6.8	426	1066	<1	4
Mo_S_	0.99	−1.2	75.5	34.1	−11.1	−4.0	−3.0	−2.9	123	631	<1	2
Mo_S2_	0.71	−1.1	8.8	28.6	−13.3	−3.6	−4.3	−6.8	164	902	10	<1

Carrier types ‘*e*’ and ‘*h*’ denote ‘electron’ and ‘hole’ for the original conduction and valence bands in perfect MoS_2_ monolayer, and ‘*d*-*e*’ and ‘*d*-*h*’ represent those for the defect states, respectively. m* is carrier effective mass and *E* is the deformation potential. All mobilities are estimated based on the value for the electron mobility in the perfect monolayer of 410 cm^2^ V^−1^ s^−1^ (ref. 48). The change of elastic moduli of perfect and defective samples is so small, which can be safely neglected. Each defective monolayer consists of only one type of defects (either vacancy or antisite) in a 6 × 6 supercell, close to an equivalent defect density revealed in the statistics.
