# Supplementary material for: Exploring atomic defects in molybdenum disulphide monolayers
Source: Nat Commun. 2015 Feb 19;6:6293. doi: 10.1038/ncomms7293 (PMC4346634; doi:10.1038/ncomms7293)
Supplement: Supplementary Information — Supplementary Figures 1-18, Supplementary Tables 1-2, Supplementary Notes 1-3 and Supplementary References [file ncomms7293-s1.pdf]

Supplementary figures

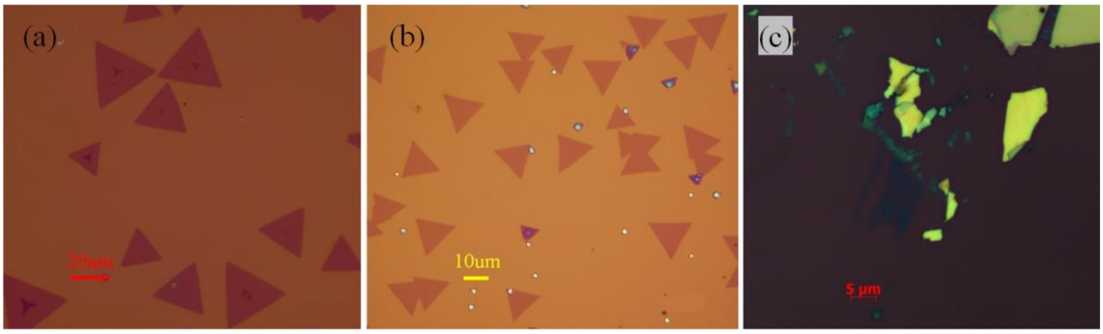

Supplementary Figure 1 | Optical microscope images of MoS_2 monolayers prepared from different methods. (**a**) Chemical vapor deposition (CVD), (**b**) Physical vapor deposition (PVD), (**c**) Mechanical exfoliation (ME).

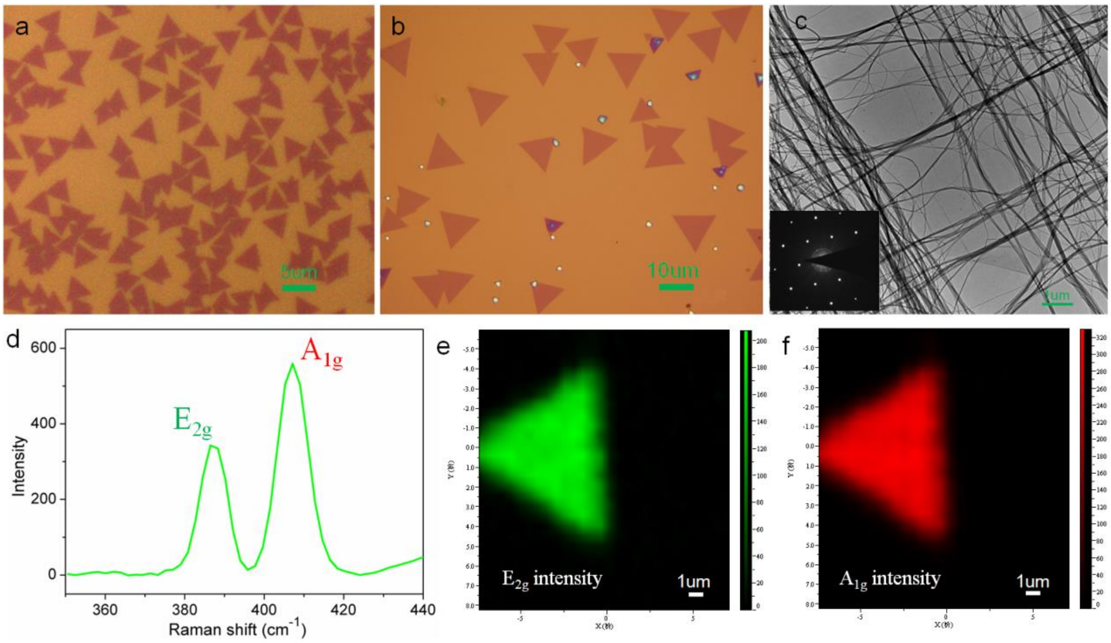

Supplementary Figure 2 | Morphology and quality evaluation of PVD MoS_2 monolayers. (**a-b**) Optical images of triangular monolayers with different sizes. (**c**) Low-magnification TEM image of transferred samples (supported on a CNT grid), with an inset of corresponding electron diffraction pattern showing the primarily single crystalline nature. (**d-f**) Raman spectroscopy and (highly uniform) Raman mapping of PVD monolayer to demonstrate its crystalline quality.

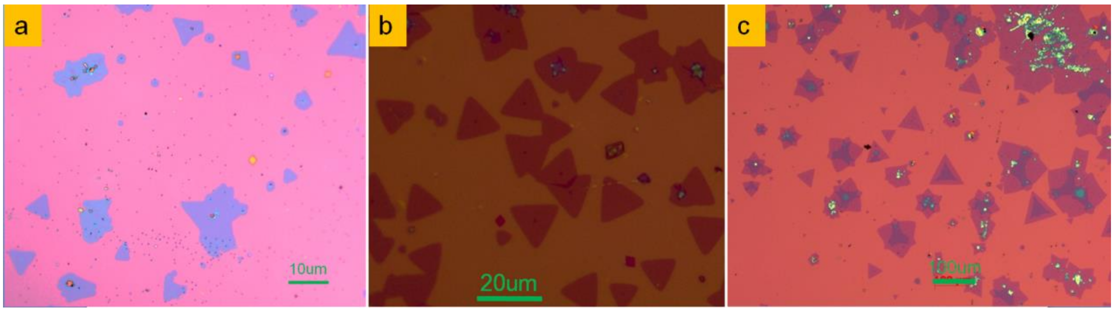

Supplementary Figure 3 | Optical images of the CVD MoS_2 monolayers. prepared at different growth temperatures (**a**) 800°C, (**b**) 875°C, (**c**) 950°C. The well-shaped samples were chosen for further microscopy characterization.

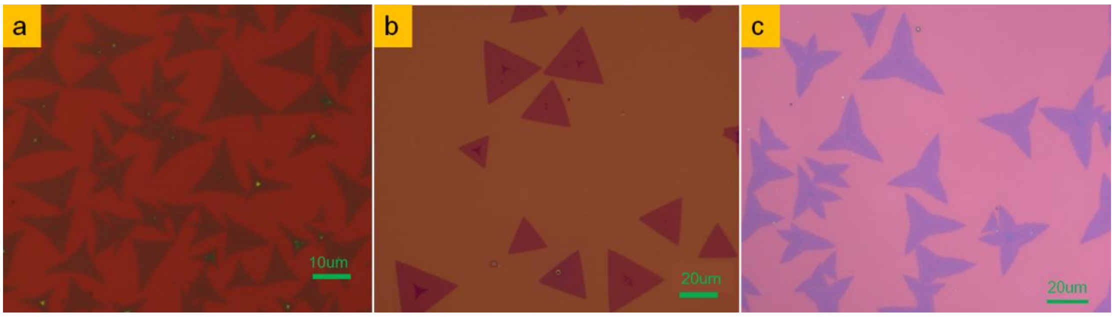

Supplementary Figure 4 | Typical optical images for the best CVD MoS_2 monolayers provided by different labs. (**a**) Samples from group A, (**b**) Sample synthesized by our group, (**c**) Samples from group B. All these samples are synthesized through the reduction of MoO_3 under S vapor flow as explained in the sample preparation part.

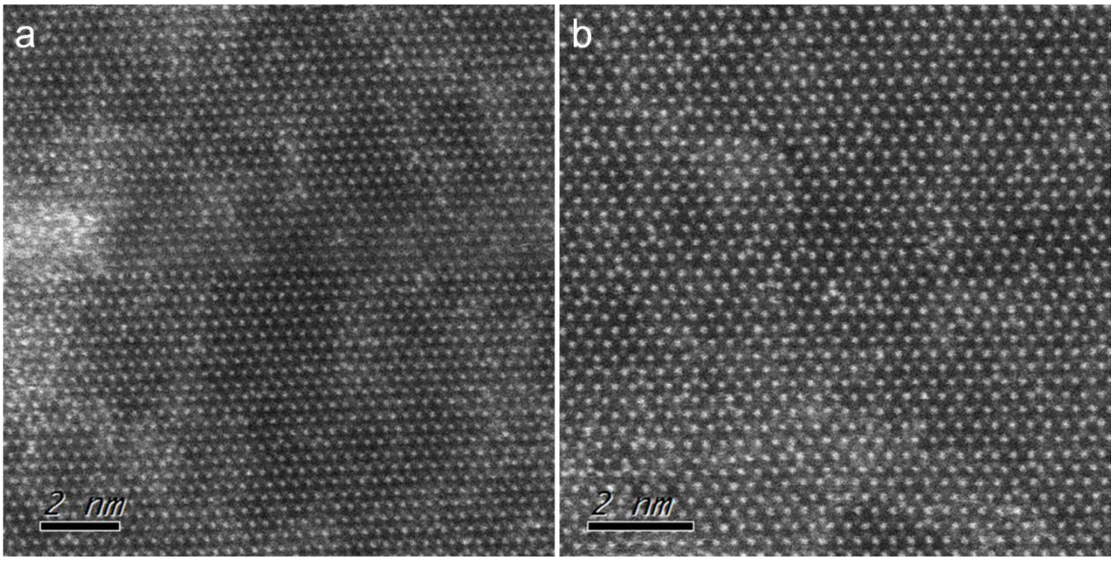

Supplementary Figure 5 | Statistical considerations of atomically-resolved ADF-STEM images. We only chose the clean areas (with little contamination) of monolayers for statistical analysis of point defects. (**a**) Heavily contaminated area of PVD monolayer. (**b**) Clean area with slight contamination on the surface, where the antisite defects can still be unambiguously resolved and counted for statistical analysis.

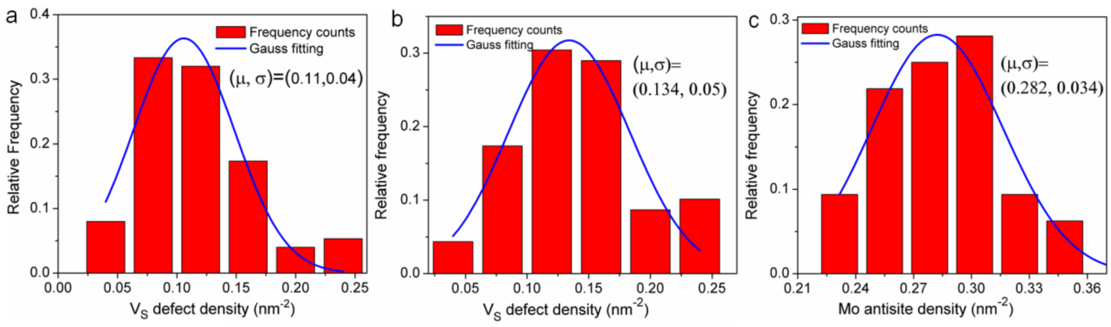

Supplementary Figure 6 | Statistical distribution of the measured density of the dominant defects in different areas of MoS_2 prepared by (a) ME, (b) CVD, (c) PVD. The statistics were done as following: for an atomic resolution ADF-STEM image recorded from a randomly chosen area, we count the number of primary defect and calculate its density; then repeat such a procedure for 70 ~ 100 ADF images of independent areas imaged, and thus obtain over 70 density values. Based on these values, we could finally draw the frequency distribution histograms, behaving like Gaussian distribution in statistics, with their mean μ and variance σ given by Gaussian fitting. For the contaminated regions, the sulfur vacancies are not distinguishable from normal S sites due to the contrast contributed by contaminated carbon. However, for many slightly and intermediately contaminated areas, Mo antisite defects are still discriminable. We checked up to 10 contaminated areas and got the defect density varying from 0.13 nm^{-2} to 0.38 nm^{-2} with an averaged density of Mo antisite of 0.22 nm^{-2}, which is close to the statistical value shown in **c**.

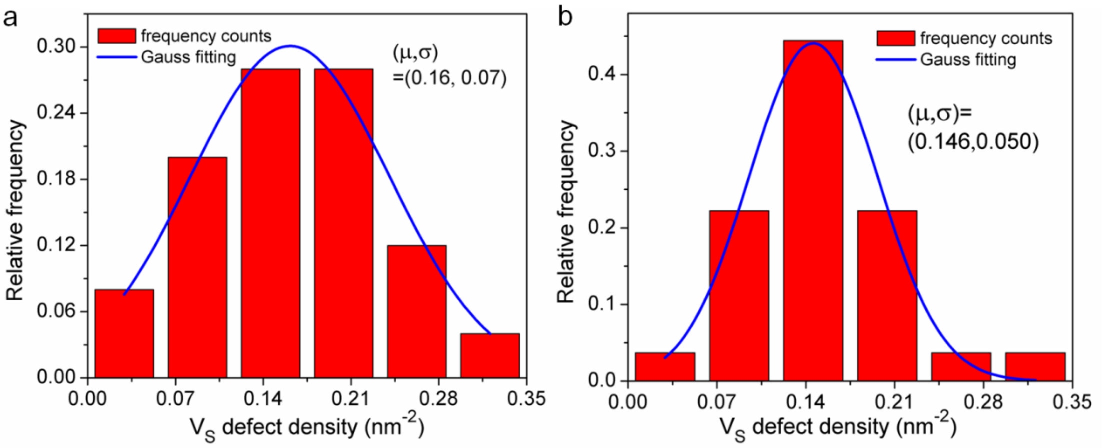

Supplementary Figure 7 | Statistics of the density of defect V_S of the "best" CVD samples from different labs. (**a**) group A and (**b**) group B. These statistics are both based on over 50 ADF-STEM images. Our experimental observations of CVD monolayers from different labs consistently demonstrate the primary defects are all S vacancies. The defect densities are all shown on the order of 0.14 ~ 0.16 nm^{-2}.

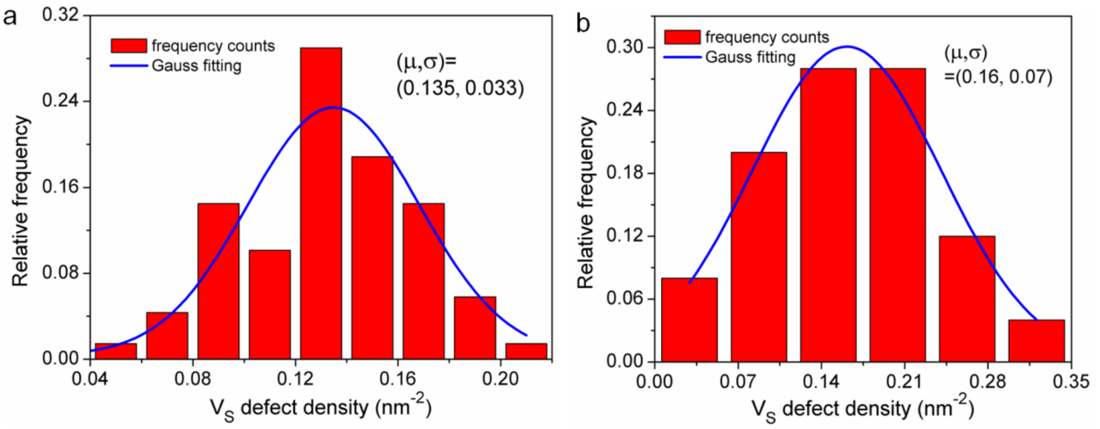

Supplementary Figure 8 | Effect of 200℃ annealing on the defect density. The statistics (**a**) before annealing, (**b**) after annealing. Our statistics shows a slightly increase of the density of sulfur vacancies owing to possible sublimation caused by annealing.

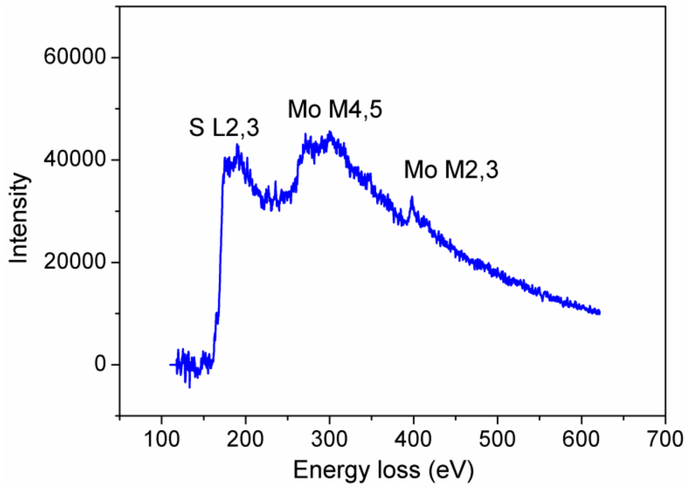

Supplementary Figure 9 | Electron energy loss spectrum of ME monolayer MoS_2, with obvious core loss of S L edge and Mo M edges.

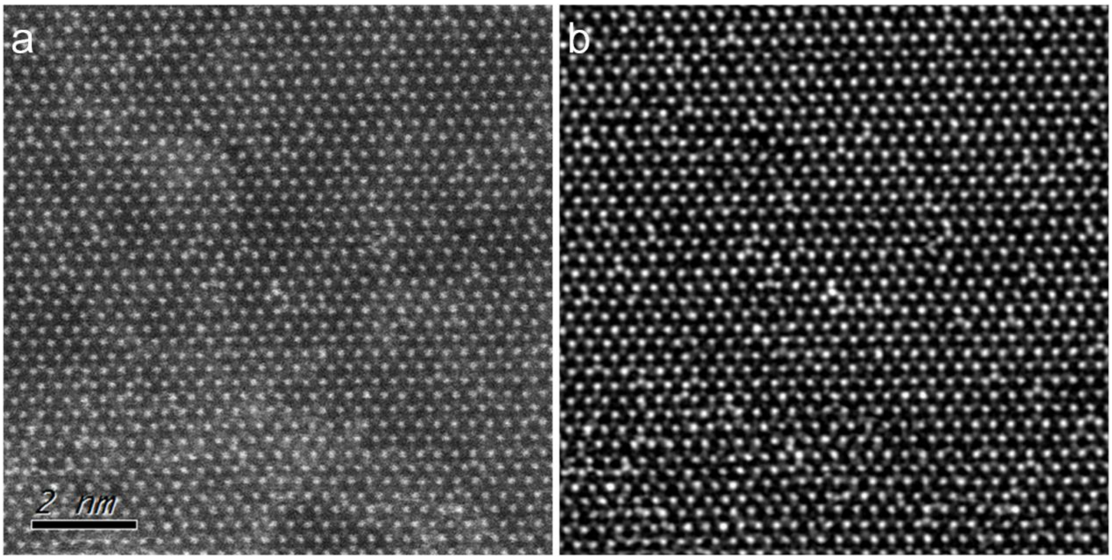

Supplementary Figure 10 | Imaging processing by Wiener filtering. (**a**) Pristine ADF-STEM images. (**b**) ADF-STEM image after Wiener filtering.

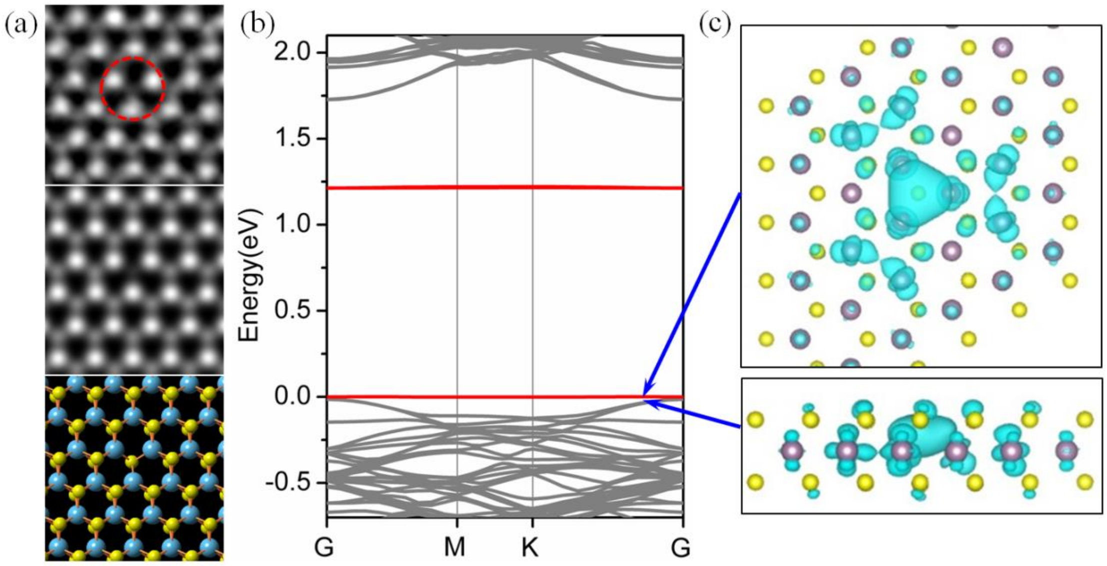

Supplementary Figure 11 | Atomic and electronic structure of V_S. (**a**) Experimental (up) and simulated (middle) ADF image of V_S, and its relaxed atomic structure (down). The simulated image is highly consistent with the experimental image. (**b**) Electronic band structure of V_S, showing localized defect states within the intrinsic gap. The energy zero point is shifted to the energy of VBM. (**c**) real-space distribution (top view and side view) of the wave function of the defect state near the energy zero, which shows the localized state within a spatial range of ~ 6 Å.

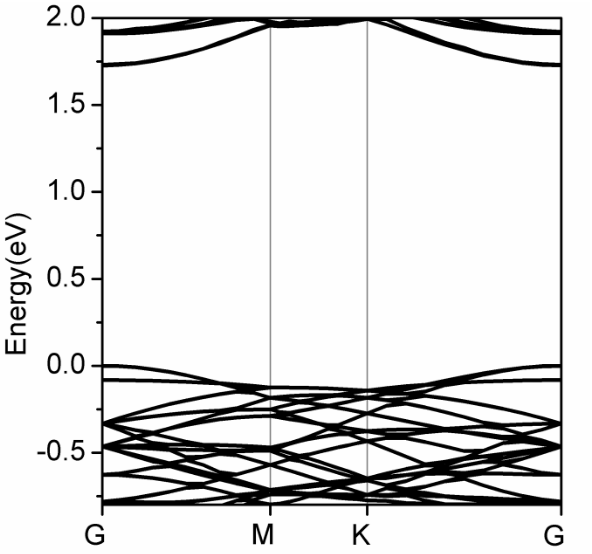

Supplementary Figure 12 | Band structure of perfect monolayer MoS_2. There is a direct gap of 1.73 eV.

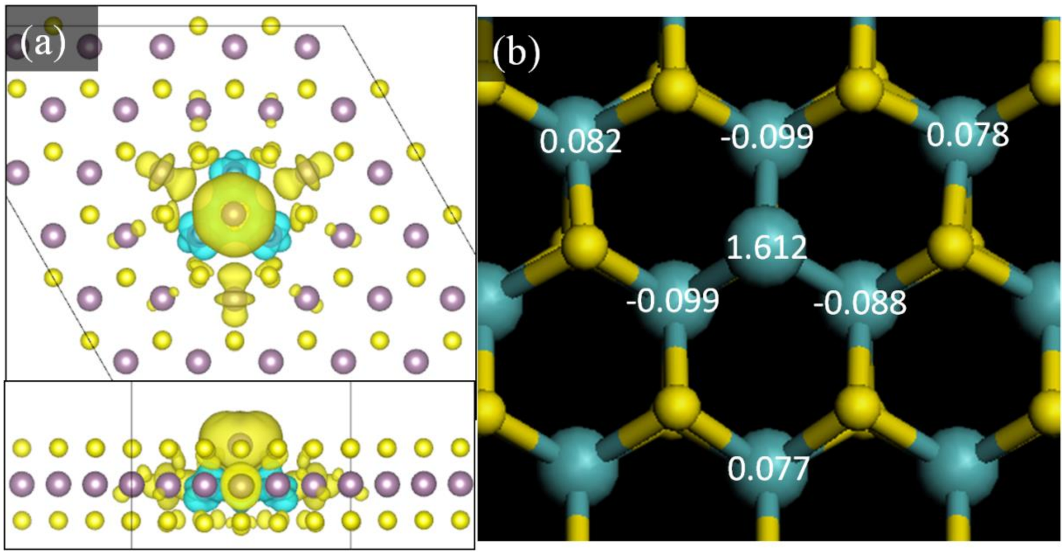

Supplementary Figure 13 | Magnetic distribution of antisite Mo_S. (**a**) Real-space distribution (top view and side view) of the total spin state of antisite Mo_S, showing the magnetic structure. (**b**) Quantitative magnetic moment distribution of antisite Mo_S, where the tiny contribution from nearby S atoms can be ignored. The unit is Bohr magneton μ_B .

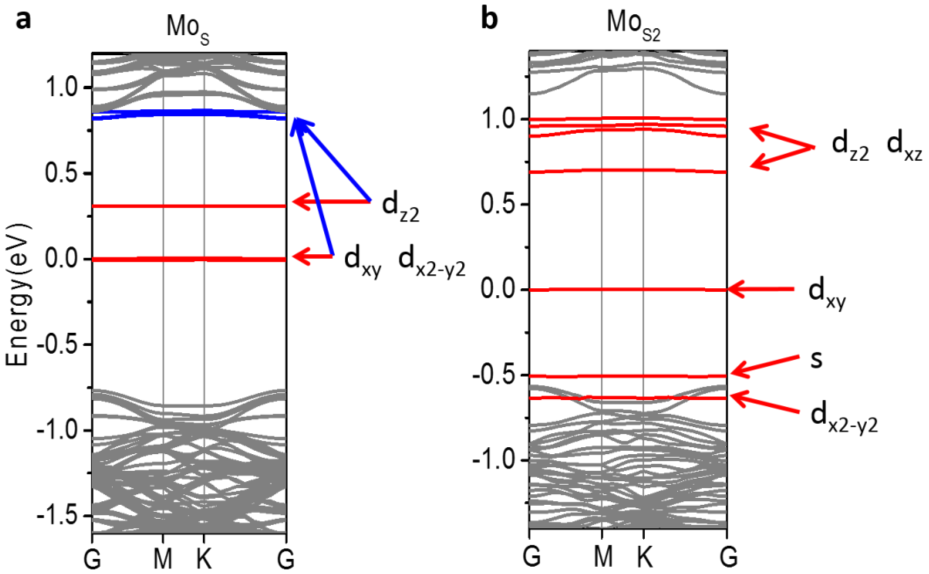

Supplementary Figure 14 | Origin of Magnetism for antisite Mo_S. Spin resolved band structures of antisites Mo_S **(a)** and Mo_{S2} **(b)**. Two spin components are shown by red and blue colors in **(a)** where it shows explicitly orbitals d_{xy} and $d_{x2\text{-}y2}$ are half filled and orbital d_{z2} is completely unoccupied. For Mo_{S2} **(b)**, however, defect orbitals, in red, are fully occupied or empty.

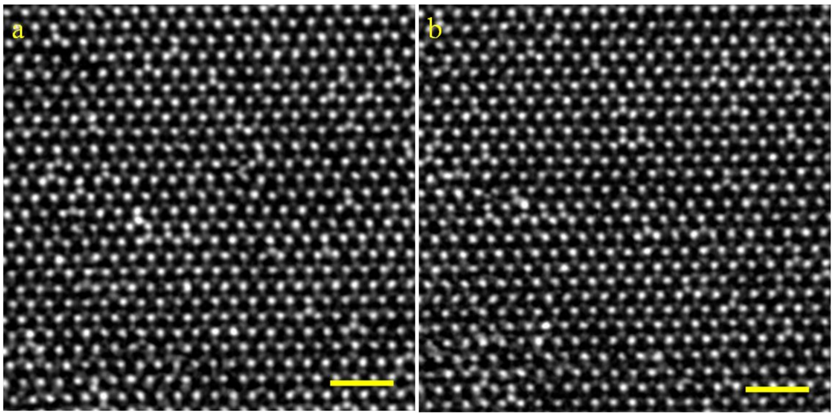

Supplementary Figure 15 | Large-area high resolution ADF-STEM images of PVD MoS_2 monolayer, which shows the native antisite defects. Scale bar: 1 nm. Note that there are no Mo vacancies nearby.

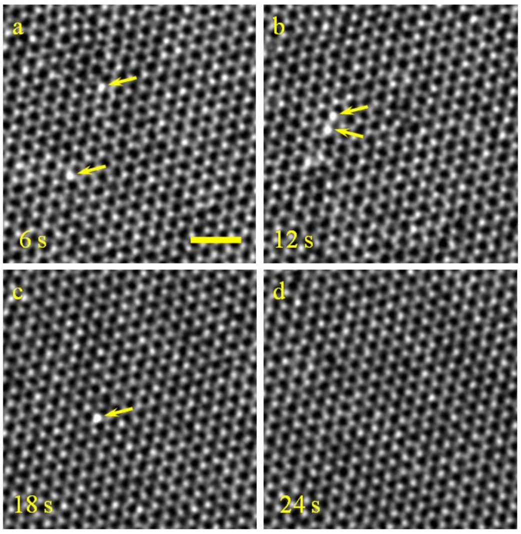

Supplementary Figure 16 | Migration of Mo adatoms on the monolayer's surface. (a-d) shows Mo adatoms were located at Mo sites and seldom move onto S sites, which indicate Mo-antisite defects are unlikely to be formed through Mo adatoms occupying S sites. Scale bar: 1 nm.

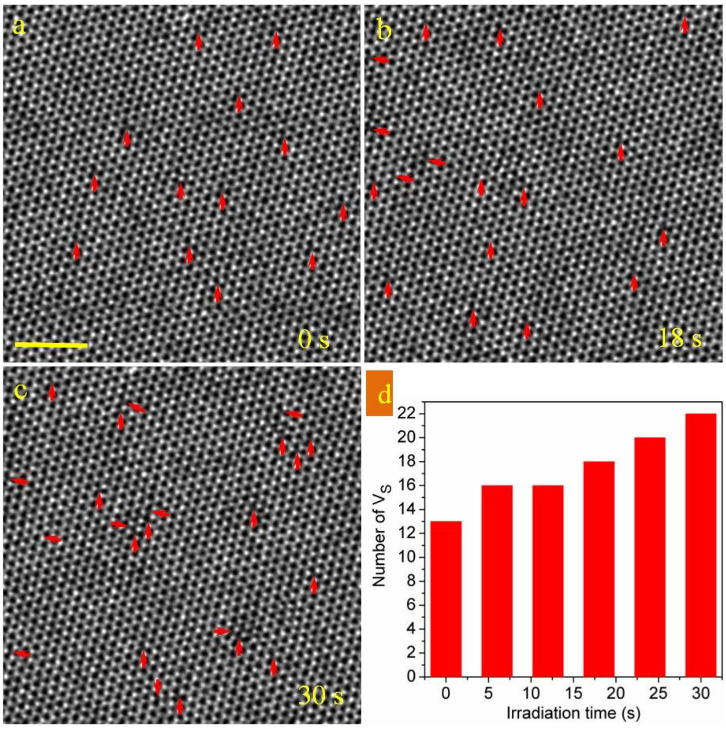

Supplementary Figure 17 | Beam radiation effect on S vacancy (V_S). (**a-c**) In situ high resolution ADF imaging at a probe current 70 pA. Red arrows indicate S vacancies. Scale bar: 2 nm. The observed sizes are all 10nm × 10nm. (d) Evolution of vacancy numbers as the beam irradiation time. A native density of V_S can be deduced to be close to 0.1 nm^{-2}.

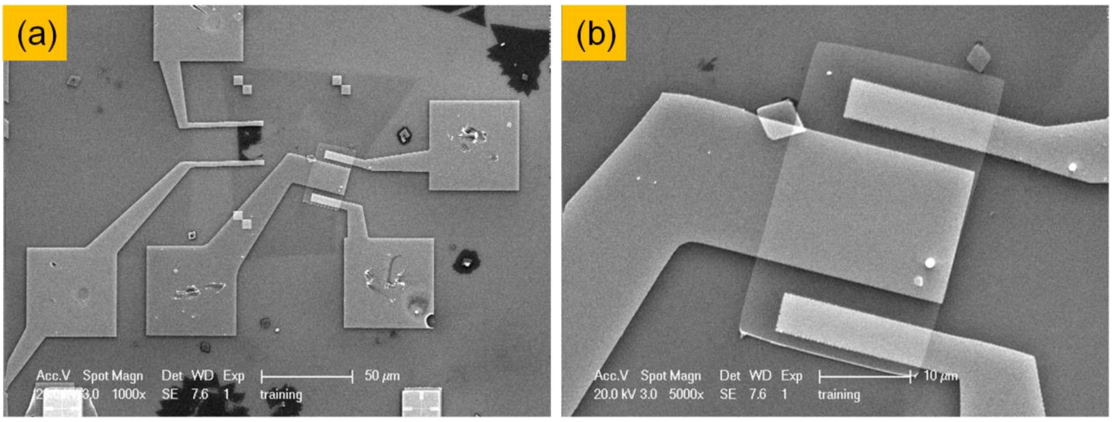

Supplementary Figure 18 | Architecture of MoS_2 based Field effect transistor (FET). (**a-b**) SEM images of MoS_2 FET at two different magnifications.

Supplementary tables

Supplementary Table 1 | Definition of point defects including vacancies and antisites.

defects	definition
V_S	Single S atom missing at the normal S2 site
V_{S2}	Double S atoms missing at one normal S2 site
V_{2S2}	Two V_{S2} appearing at two neighboring S2 sites
V_{Mo}	Single Mo atom missing at normal Mo site
V_{MoS6}	Central Mo and six neighboring S atoms missing
S_{Mo}	Single S atom replacing the normal Mo site
$S2_{Mo}$	Double S atoms replacing one normal Mo site
Mo_S	Single Mo atom replacing only one S atom at one normal S2 site
Mo_{S2}	Single Mo atom replacing both S atoms at one normal S2 site
$Mo2_{S2}$	Two Mo atoms replacing both S atoms at one normal S2 site

Supplementary Table 2 | Comparison of the advantages and disadvantages of different methods for synthesis of atomically thin layers.				
Methods	Lateral size	Surface cleanness	Intrinsic defects density	Production efficiency
ME	<10 µm	clean	quite low	low
Liquid-phase exfoliation[1, 2]	<5 µm	Much contamination	high	low
CVD	20~200 µm	Less contamination	moderate	highest
PVD	5~30 µm	Less contamination	high	high
The green items indicate good quality meeting the criteria for electronic application. If in pursuit of excellent electronic performance, ME will be the best choice. If in pursuit of wafer-scale electronic and optoelectronic application, CVD is a proper way to meet the moderate requirements such as high resolution display and power devices.				

Supplementary notes

Supplementary Note 1 Sample preparation and transfer. ME-MoS_2 monolayers were prepared by mechanical cleavage of natural bulk crystal (SPI supplies). The monolayer regions on the exfoliated thin flakes can be directly identified from their optical contrast under an optical microscope (Zeiss A2m), and then transferred for ADF-STEM observations as following: firstly a copper TEM grid was placed onto the specimen with the holey carbon film side facing the selected monolayer region, then a drop of isopropanol (IPA) was deposited to strengthen the contact between the TEM grid and the substrate via the sublimation of IPA. In the next step, potassium hydroxide solution was introduced to etch the underneath silica substrate, making thin flakes float and adhere onto the carbon film. The TEM grid containing the transferred MoS_2 thin flakes was immersed into the distilled water to wash off surface inorganics for several minutes. Finally, this TEM grid was dried in IPA. No polymers like PMMA was used during the whole process, which

largely reduced the contaminations.

CVD monolayers were synthesized through the reduction of precursor MoO_3 by sulfur vapor flow at ambient pressures following the previously reported method[3,4]. Most of the CVD specimens were synthesized as the following optimized condition developed by the ZJU group: the temperatures for the sublimation of MoO_3 and the growth of MoS_2 monolayers were both set to 850 °C. The silicon substrates with 300 nm-thick SiO_2 capping layers were placed above the MoO_3 precursors (Aladdin, 99.9 %) with the oxides layers facing downward during the growth. The sulfur source (Aladdin, 99.999 %) was placed at the upstream in a separated furnace, which was heated to 180 °C and introduced into the main furnace by Argon gas with a flow of 50 sccm after the MoO_3 precursors were heated to 850 °C. The growth time was typically set to 15 minutes.

As reported in Ref 5, PVD MoS_2 monolayers used in this study were synthesized by thermal evaporation of MoS_2 powders (Sigma-Aldrich, 99 %) at a temperature of 950 °C. Ar (2 sccm) and H_2 (0.5 sccm) were used as the carriers gases. The pressure of the growth chamber was about 8 Pa, and the growth time was usually 10 minutes.

PVD and CVD monolayers were transferred onto the TEM grid as following: firstly the SiO_2 substrates with monolayer samples were covered with PMMA film after spin coating, and then dried in air at 120 °C for 5 minutes. The substrates were immersed the boiling NaOH solution (1 mol/L) which was heated up to 200 °C to etch away the underneath SiO_2 layers. The floating PMMA film was picked up with a clean glass slide, and then transferred into the distilled water for several cycles to wash away surface residues. In the next step, the PMMA film was lifted out by a TEM grid covered with lacey carbon film, and then dried naturally in ambient. This TEM grid was heated at 120 °C for 5 minutes in air before immersed into hot acetone for about 24 hours to remove the PMMA.

Supplementary Note 2 Discussion on the origin of magnetism of antisite defects. The presence of magnetic moment in antisite Mo_S can be well explained by crystal field theory and hybrid orbital theory. The antisite Mo takes d^4s hybridization forming five orbitals, i.e. s, d_{xz}, d_{yz}, d_{xy} and $d_{x2\text{-}y2}$, which are filled by eight electrons, six from the anti-site Mo and two from the adjacent Mo atoms (Supplementary Figure 14). The former three are occupied by six electrons and the

latter two, degenerated in energy, are filled by two electrons which are unpaired and their spin direction aligns in parallel, due to on-site Coulomb repulsion as described by Hund's Rules. In terms of antisite Mo_{S2}, however, the antisite Mo atom is significantly off the center of the three neighboring Mo atoms, leading to orbital d_{xz} missing in the formation of bonding, therefore, the antisite Mo takes d^3s hybridization forming four hybridized orbitals, originated from s, d_{xy}, d_{x2-y2}, d_{yz}, filled by eight electrons. As a result of the d^3s hybridization, the defect Mo_{S2} is non-magnetic. It might be possible in the future to directly measure the magnetic properties associated with this specific antisite defects in MoS_2 monolayer samples by using spin-polarized scanning tunneling microscopy[12].

Supplementary Note 3 Beam effect on point defects. The definition of each point defect is listed in Supplementary Table 1. Point vacancies in monolayer have been reported elsewhere by several groups[7-9], and theoretical and experimental findings[10,11] are plentiful in terms of the electronic properties. However, systematic investigation on antisite defects has not been reported yet and public profound awareness about their effect on electronic properties still lacks. The detailed atomic structures of all antisite defects are shown in Fig. 2, with the top view and side view of the abnormal sites to demonstrate the atomic scale structure relaxation. The observed antisite defects are believed to be native to the specimen, not induced by electron beam irradiation. This is because the formation of a Mo_S antisite defect as a result of beam irradiation involves two steps including the emerging of V_S and the occupying of Mo atom (from nearby Mo lattice or Mo adatom) which are not energetically favorable. Even if sulfur vacancy can be generated under electron beam, Mo atom nearby is unlikely to jump into S vacancies to become antisite Mo_{S2} or Mo_S unless Mo vacancy is also formed (Supplementary Figures 15, 16). The experimental fact that very few Mo vacancies appear and that almost no Mo adatoms evolve into antisite defects confirms Mo_S and Mo_{S2} to be intrinsic defects. It should be noted the statistical concentration of sulfur vacancies may be slightly overestimated due to the beam damage even if the microscope works at low accelerating voltage (Supplementary Figure 17). But the measured concentration of antisite defects almost totally reflects its intrinsic density, and shall not be overstated.

Supplementary references

1. Xiao, J. *et al*. Exfoliated MoS_2 nanocomposite as an anode material for lithium ion batteries. *Chem. Mater.* **22**, 4522-4524 (2010).
2. Hwang, H., Kim, H. & Cho, J. MoS_2 nanoplates consisting of disordered graphene-like layers for high rate lithium battery anode materials. *Nano Lett.* **11**, 4826 (2011).
3. Lee, Y.-H. *et al*. Synthesis of large-area MoS_2 atomic layers with chemical vapor deposition. *Adv. Mater.* **24**, 2320-2325 (2012).
4. Najmaei, S. *et al*. Vapour phase growth and grain boundary structure of molybdenum disulphide atomic layers. *Nat. Mater.* **12**, 754-759 (2013).
5. Wu, S. *et al*. Vapor-solid growth of high optical quality MoS_2 monolayers with near-unity valley polarization. *ACS Nano* **7**, 2768-2772 (2013).
6. Feng, Q. *et al*. Growth of large-area 2D $MoS_{2(1-x)}Se_{2x}$semiconductor alloys. *Adv. Mater.* **26**, 2648-2653 (2014).
7. Komsa, H.P., Kurasch, S., Lehtinen, O., Kaiser, U. & Krasheninnikov, A.V. From point to extended defects in two-dimensional MoS_2: evolution of atomic structure under electron irradiation. *Phys. Rev. B* **88**, 035301(2013).
8. Komsa, H.P. *et al*. Two-dimensional transition metal dichalcogenides under electron irradiation: defect production and doping. *Phys. Rev. Lett.***109**, 035503 (2012).
9. Zhou, W. *et al*. Intrinsic structural defects in monolayer molybdenum disulfide. *Nano Lett.* **13**, 2615-2622 (2013).
10. Qiu, H. *et al*. Hopping transport through defect-induced localized states in molybdenum disulphide. *Nat. Commun.* 4:2642 doi: 10.1038/ncomms3642 (2013).
11. Zhu, W. *et al*. Electronic transport and device prospects of monolayer molybdenum disulphide grown by chemical vapour deposition. *Nat. Commun.* 5:3087 doi: 10.1038/ncomms4087 (2014).
12. Wiesendanger R. Spin mapping at the nanoscale and atomic scale. *Rev. Mod. Phys.* **81**, 1495 (2009).
